# Dataset on the impacts of sand and leaf litter on the hydrological performance of green roofs as surrogate for infiltration-based blue-green infrastructure (BGI)

**DOI:** 10.1016/j.dib.2025.111337

**Published:** 2025-01-28

**Authors:** Prabhat Joshi, Juan Naves, Jose Anta, Max Maurer, João P. Leitão

**Affiliations:** a Institute of Environmental Engineering (IfU), ETH Zürich, Laura-Hezner-Weg 7, 8093 Zürich, Switzerland; b Department of Urban Water Management (SWW), Eawag, Überlandstrasse 133, 8600 Dübendorf, Switzerland; c Universidade da Coruña, Water and Environmental Engineering Research Team (GEAMA), Centre for Technological Innovation in Construction and Civil Engineering (CITEEC), Campus de Elviña, 15071 A Coruña, Spain

**Keywords:** Clogging, Detention, Infiltration, Maintenance, Rainfall generator, Retention, Surface sealing

## Abstract

This dataset contains raw and processed data from controlled experiments conducted in a rainfall simulator to quantify the impacts of incremental sand and leaf litter accumulation on the hydrological performance of blue-green infrastructure (BGI). The tests were conducted in a controlled indoor environment using two BGI boxes (approximately 3.84 m² each), representing a typical infiltration-based BGI setup with vegetation, a 6-cm deep substrate, and storage layers. Soil moisture sensors and tipping buckets were installed to measure underdrain flow. In one box, sand was incrementally added (2–18 kg.m^−^²); in the other, leaf litter accumulation ranged from 0.3 to 1.725 kg (total). Each scenario received rainfall (16.66 mm.h^−1^ for Box 1; 18.66 mm.h^−1^ for Box 2) for 30 minutes, with intervals larger than four hours between tests. The dataset can be used to understand the impact of shock events that introduce high pollutant loads to the BGI surface and subsurface, affecting their hydrological performance. It can also be used to study the maintenance needs of BGI to sustain their hydrological functionality.

Specifications Table

**Table:** 

Subject	Environmental Science
Specific subject area	Hydrological performance deterioration of blue-green infrastructure (BGI) due to poor maintenance
Type of data	Table, Image, Chart, Graph, Figure
Data format	Raw, Analysed, Processed
Data collection	Two BGI boxes, comprising HYDROPACK® trays^i^, Vegetal i.D.®, were subject to different amounts of sand and leaves and rainfall with constant intensity (Box 1: 16.66 mm.h^−1^; Box 2: 18.66 mm.h^−1^) and duration (30 min). Underdrain flow was measured using HyQuest Solutions' single tipping bucket flow gauges (TB0.5L)^ii^) as binary signals logged as “0” or “1” (tip) every 3s. The signal was smoothed, aggregated to 1-min, and converted to flow rates in mm.h^−1^. TEROS 12iii capacitance sensors (two in Box 1 and one in Box 2) recorded soil moisture at 5-min intervals, the data of which was adjusted by calibration and resampled to 1-min.
Data source location	Institution: Centre for Technological Innovation in Construction and Civil Engineering (CITEEC), Universidade da Coruña (UDC) City/Town/Region: 15071 A Coruña, Spain Country: Spain Latitude and longitude: 43° 21′ 44.4348′' N and 8° 24′ 41.5440′' W
Data accessibility	Repository name: ERIC/Open Data identification number: https://doi.org/10.25678/000DDP Direct URL to data: https://opendata.eawag.ch/dataset/bgi-maintenance-experiment
Related research article	None

i HYDROPACK®green roof trays, Vegetal i.D.®: https://www.vegetalid.com/solutions/green-roofs/the-all-in-one-system-hydropack.html.

ii HyQuest Solutions’ single tipping bucket flow gauges (TB0.5L): https://www.hyquestsolutions.com.au/products/hardware/water-flow/tb1l-tb05l-tipping-bucket-flow-gauge#tab=description.

iii TEROS-12, METER group: https://www.metergroup.com/en/meter-environment/products/teros-12-soil-moisture-sensor.

## 1 Value of the Data

• The presented data show the extent to which the BGI underdrain flow volume and hydrological characteristics change when these infrastructures are subjected to different amounts of surface sealing and clogging;
• The data could be used to understand the influence of shock events that bring high amounts of pollutant loads on the BGI surface/sub-surface, which affect their hydrological performance;
• These data can be used to support or validate models that simulate the influence of incremental sediment/pollutant build-up;
• The obtained data also shed light on the maintenance requirements of BGI to preserve their hydrological performance during their lifespan.

## 2 Background

BGI systems retain and detain [1] stormwater that, in turn, helps mitigate pluvial flooding [2] and combined sewer overflows [3,4]. Therefore, these infrastructures are increasingly being adopted in cities worldwide. Many studies have focussed on BGI planning and designing strategies (e.g., [[5], [6], [7]]). However, like any other urban water infrastructure, BGI also requires regular monitoring and maintenance to preserve its hydrological performance, which has been overlooked and understudied in published literature [8,9]. Inadequately maintained BGI can result in surface sealing and substrate clogging that will yield reduced hydrological benefits [10], yet the extent of the degradation is scarce in the published literature. This research gap was the motivation to conduct controlled tests to quantify the effects of BGI subject to different amounts of sediments – represented by incremental sand and leaf litter accumulation – and relate the load to the decline in the hydrological performance.

## 3 Data Description

### 3.1 Organisation of files and folders

**Fig. 1:**
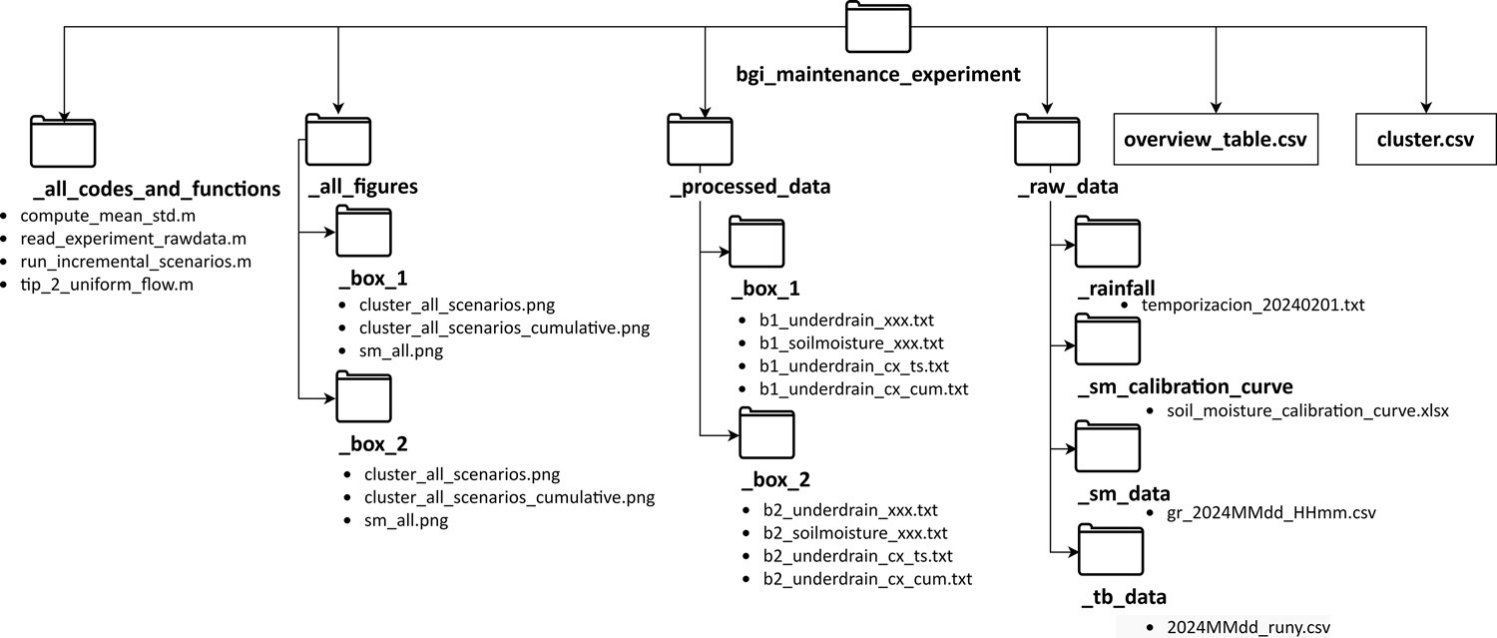
Overview of the files and folder structure in the repository.

The dataset contains a main folder called “bgi_maintenance_experiment” with four sub-folders and two comma-separated values (CSV) files (Fig. 1). All the codes and functions used to process the raw data and plot the processed data are presented in the “_all_codes_and_functions” folder. The raw data, comprising rainfall data, soil moisture calibration curve, soil moisture data, and tipping bucket data, are in the “_raw_data” folder. The processed data are stored in the “_processed_data” folder and the figures in the “_all_figures” folder. The “overview_table.csv” lists all the scenarios (Table 1) for the two boxes, whereas the “cluster.csv” file contains information for the visualisation of the results.

**Table 1:** Content of the "overview_table.csv" file. The variables in the italics differ for different test runs.

Column name	Description
Scenario	Experiment number
Cluster_B1	Cluster number of similar experiment types (scenarios) for Box 1
Cluster_B2	Cluster number of similar experiment types (scenarios) for Box 2
Time	Date and time of the experiments
Scenario_name_B1	Scenario name for the experiments conducted in Box 1
Sc_B1	Cluster name for the similar experiments conducted in Box 1
Scenario_name_B2	Scenario name for the experiments conducted in Box 2
Sc_B2	Cluster name for the similar experiments conducted in Box 2
TB_filename	Name of the raw tipping bucket file (filename example: 2024*MMdd*_run*y*.csv)
SM_fileaname	Name of the raw soil moisture file (filename example: gr_2024*MMdd*_*HHmm*.csv)
rainFile	Rainfall time series data (filename: temporizacion_20240201.txt)
Remarks	Comments about the experiments

### 3.2 Workflow

**Fig. 2:**
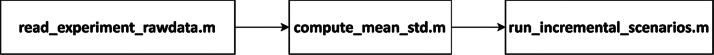
Workflow diagram.

Fig. 2 displays the workflow diagram to generate processed data and plots from the raw data, which has three code files developed using Matlab R2022a (version 9.12). The dependencies, input, requirements, and outputs for each code file are presented in Table 2. The “read_experiment_rawdata.m” takes raw rainfall, soil moisture, and tipping bucket signals from the two boxes and generates processed data in 1-min resolution for each scenario run (listed in the overview table). The “compute_mean_std.m” rereads the overview table for clustering tests with similar scenarios for the two boxes. For example, for the two boxes, three tests were carried out for the “Reference” case in which no pollutants were added, from which the output files were clustered to determine their mean and standard deviation for further analysis and visualisation. The “run_incremental_scenarios.m” reads the mean and standard deviation of the clustered data (rainfall, soil moisture, and tipping bucket) for the two boxes and generates the plots.

**Table 2:** Dependencies, inputs, required files, and outputs of the code files in the workflow.

File	Dependencies (*function*)	Input	Required files/data	Output
read_experiment_ rawdata.m	• Statistics and Machine Learning Toolbox (*polyval*)	• Path to the main folder (bgi_maintenance_experiment)	• overview_table.csv • raw rainfall file (temporizacion_20240201.txt), • raw soil moisture file (gr_2024MMdd_HHmm.csv), • raw tipping bucket file (2024MMdd_runy.csv) • tip_2_uniform_flow.m	• smoothed/processed underdrain flow file (b1_soilmoisture_xxx.txt) • soil moisture file (b1_underdrain_xxx.txt)
compute_mean_std.m	• None	• Path to the main folder (bgi_maintenance_experiment) • Box number (1 and/or 2)	• overview_table.csv • smoothed/processed underdrain flow file (b1_underdrain_xxx.txt)	• mean and standard deviation of the underdrain flow with similar scenarios (cluster) (b1_underdrain_ccc_ts.txt)
run_incremental_ scenarios.m	• None	• Path to the main folder (bgi_maintenance_experiment) • Box number (1 and/or 2)	• overview_table.csv • cluster.csv • mean and standard deviation of the underdrain flow with similar scenarios (cluster) (b1_underdrain_ccc_ts.txt)	• plots of the rainfall, underdrain flow, and soil moisture data (cluster_all_scenarios.png; cluster_all_scenarios_cumulative.png; sm_all.png)

### 3.3 Overview of the processed data

**Fig. 3:**
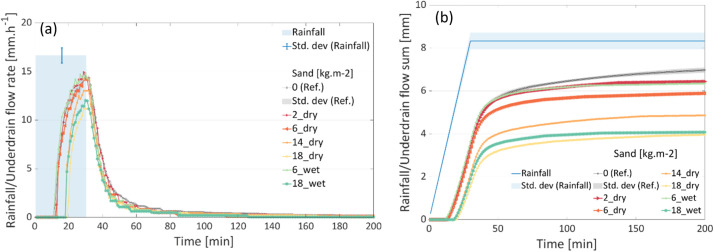
Results of the incremental sand (dry and wet) tests in Box 1. (a) time series and (b) cumulative sum.

**Fig. 4:**
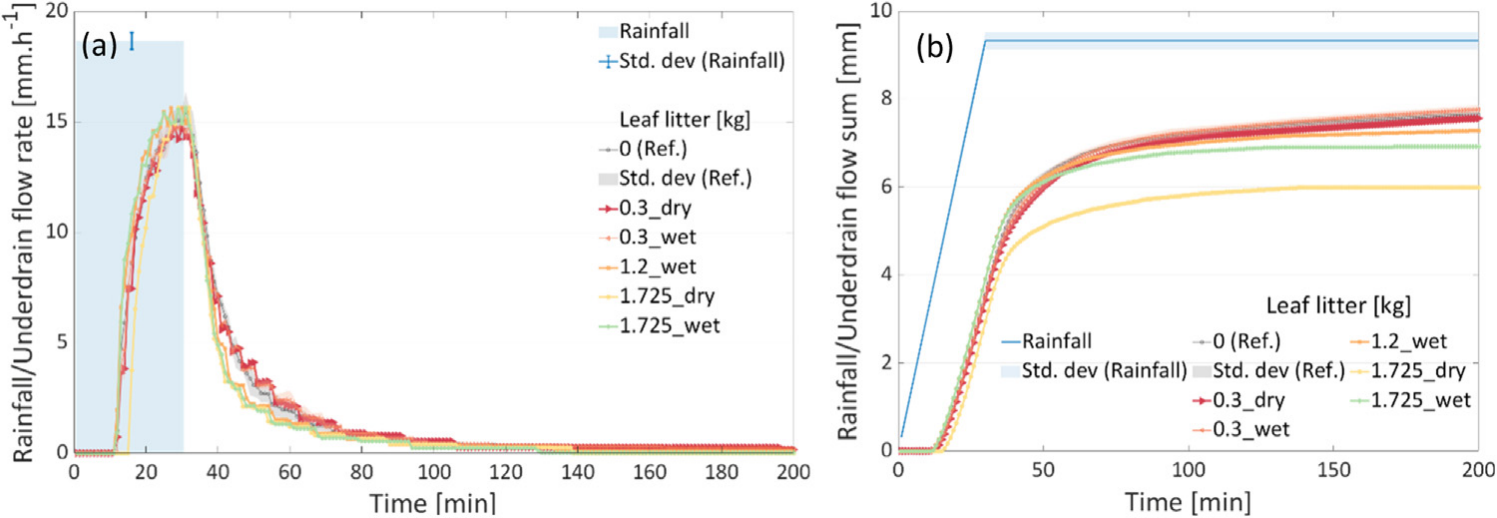
Results of the incremental leaf litter (dry and wet) tests in Box 2. (a) time series and (b) cumulative sum.

**Fig. 5:**
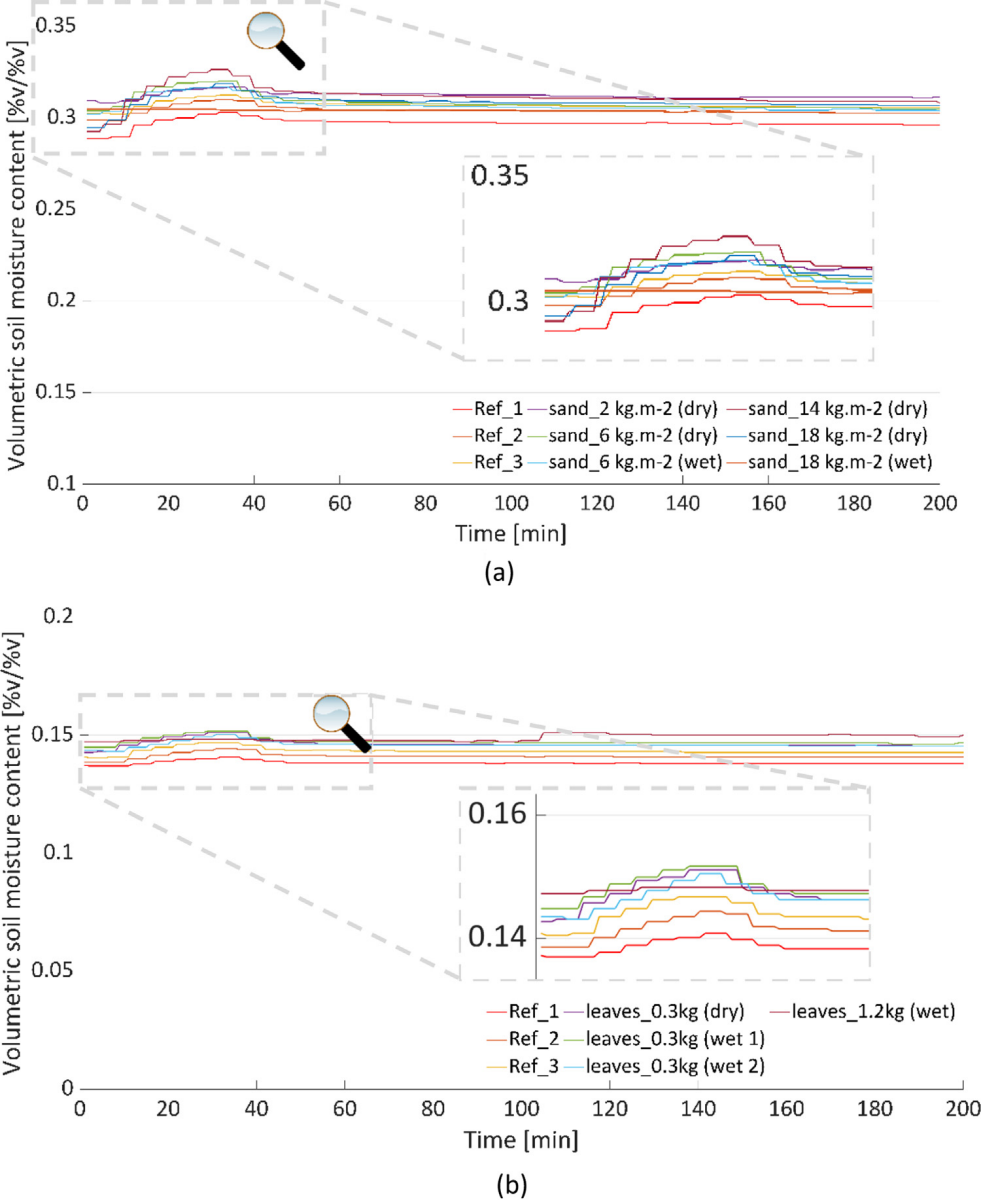
Calibrated soil moisture values for the two boxes. (a) Box 1; (b) Box 2.

The line plots of the processed data for the two boxes are stored in the “_all_figures” folder. An overview of the plots are shown in Fig. 3, Fig. 4, Fig. 5. Fig. 3(a) shows the time series of the incremental sand accumulation (for dry and wet sands) for Box 1, whereas Fig. 3(b) shows the corresponding cumulative sums. For the rainfall intensity and the underdrain flow for the “reference” condition, redundant measurements are depicted using the mean value and the standard deviation. Similarly, the plots for the incremental leaf litter scenarios from Box 2 are depicted in Fig. 4. The soil moisture values for the two boxes during the tests are presented in Fig. 5. Note that the soil moisture values for the 1.725 kg (dry and wet) leaf litter tests are not available. However, the test was not discarded, as the test conditions were similar to those for other weights.

## 4 Experimental Design, Materials and Methods Site Description

**Fig. 6:**
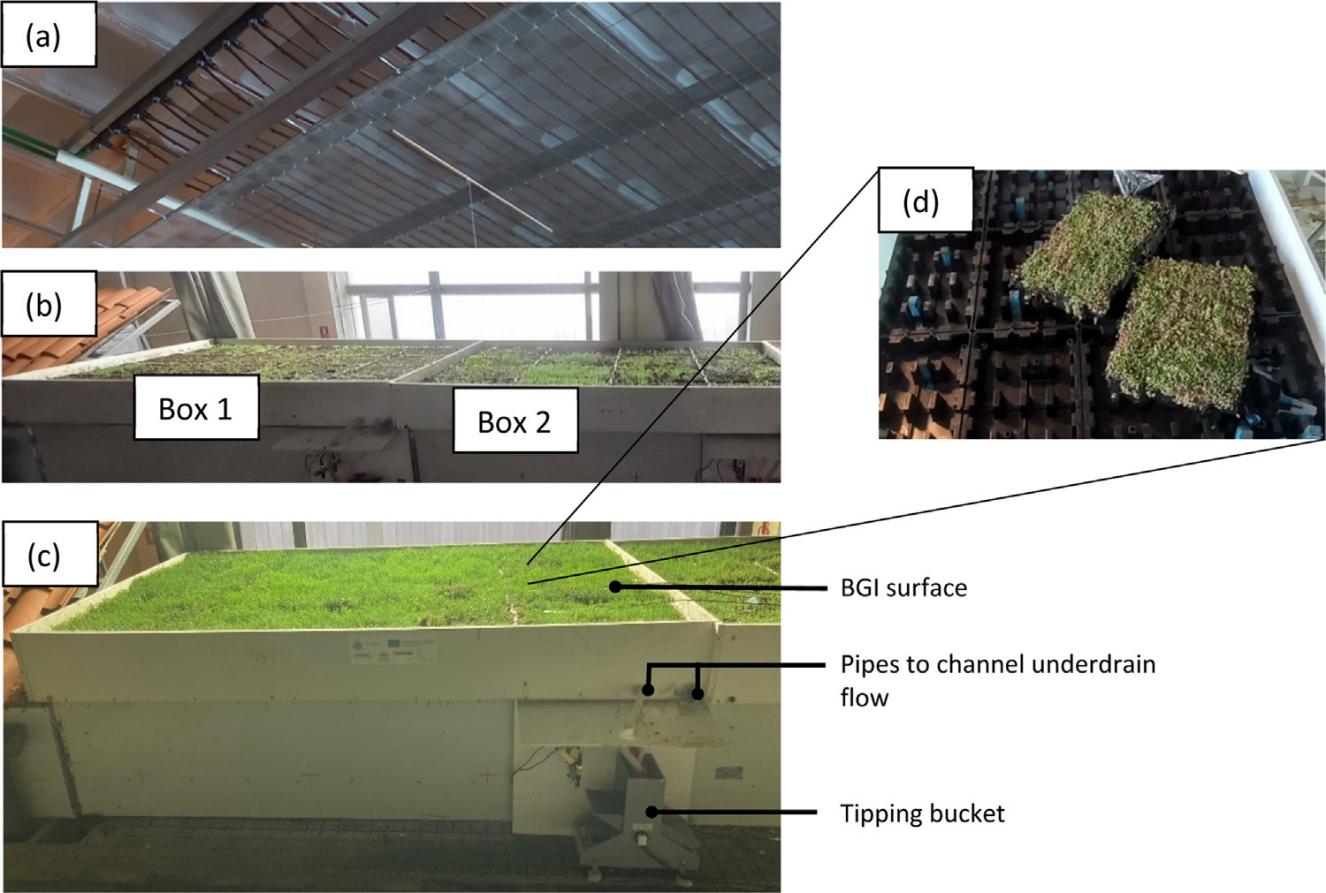
Overview of the rainfall generator and the BGI housed in boxes with tipping buckets to measure underdrain flow. (a) Rainfall generator; (b) Two BGI boxes showing the arrangement of trays; (c) Front view of one of the boxes with the tipping bucket to measure underdrain flow; (d) Tray arrangements.

All the tests were conducted at CITEEC^i^ in the University of Coruña (UDC), Spain, which has an indoor rainfall generator (Fig. 6(a)) [11], and three BGI boxes (of which only Boxes 1 and 2 (Fig. 6(b)) were used for our study). The boxes were each equipped with tipping buckets to quantify the underdrain flow (Fig. 6(c)) and soil moisture sensors to measure soil water content (not visible in Fig. 6(c)).

### 4.1 BGI arrangement

Each of the two boxes – covering 3.84 m^2^ area – comprised thirty-two HYDROPACK® trays, stacked in two layers (sixteen in each layer) housed in a plastic box at approximately 1% slope.

The top layer comprised a vegetation layer (*Sedum sp.*) atop a 6 cm deep substrate layer of pozzolans, expanded arlite and pumice stones, and a permeable membrane for the down flow of infiltrated water into the bottom layer. The top layer was sealed along the box's perimeter and the space between the trays to prevent rainfall water from passing directly, which ensured all water infiltrated through the substrate into the bottom layer. Ponding or overland flow, if any, was not measured.

The bottom layer was a storage layer comprising sixteen trays, all connected in a way that the water could flow from one tray to another to the outlet of the box. The water flowing into the bottom layer was drained onto a tipping bucket flow gauge as underdrain flow without allowing much water to be stored.

The soil moisture sensors were placed in each box (two in Box 1, one in the box's centre, another near the outlet, and one in Box 2 in the centre) to measure the soil moisture level. These sensors were calibrated by using an oven-dried soil sample, which was first wetted and then allowed to dry, all the while recording their soil moisture content. The recorded soil moisture values were compared against the weight of the sample to obtain a calibration curve.

### 4.2 Rainfall generator

A detailed description of the rainfall generator can be found in Naves *et al*. [11]. For our tests, the original water pressure of the system was reduced to the minimum level possible without compromising rainfall uniformity. This step was necessary to not limit the study to high-intensity or extreme rainfall events.

The rainfall uniformity was ensured by collecting rainwater in sixteen collection vessels on each of the two boxes for 15 minutes and comparing the weight of the collected rainwater. Furthermore, the total volume of rainfall was confirmed by removing all the trays, leaving only the empty boxes, and collecting the rainwater for 30 minutes. The sum was then divided by the total area and duration to obtain the rainfall intensity. This process was repeated thrice to obtain the mean value and the corresponding standard deviation for the two boxes: Box 1: 16.66 ± 0.78 mm.h^−1^ and Box 2: 18.66 ± 0.40 mm.h^−1^.

### 4.3 Experimental design

#### 4.3.1 No interference (Reference condition)

Tests without leaves and sand on the BGI surface were first conducted to establish the baseline (reference) condition against which the other scenarios were to be compared. To simplify the tests and ensure that the major hydrological response was only in the underdrain flow, the following choices, simplifications and assumptions were made:

• A fixed amount of rainfall (fixed intensity and duration) was supplied.
• The evapotranspiration (ET) was neglected in the analysis as the facility was in indoor conditions, which had high relative humidity.
• The sealing of trays and the relatively low rainfall intensity prevented surface overflow.
• The tests were all run in wet conditions, i.e. when the substrate was close to its field capacity. Doing so ensured that the initial soil moisture conditions were similar for all the tests and that the soil storage was minimal (and the response was discernible in the underdrain flow).
• The tests for the reference conditions were conducted three times, with an inter-rainfall event duration of four hours, to ensure redundant hydrological performance.

#### 4.3.2 Sand accumulation

**Fig. 7:**
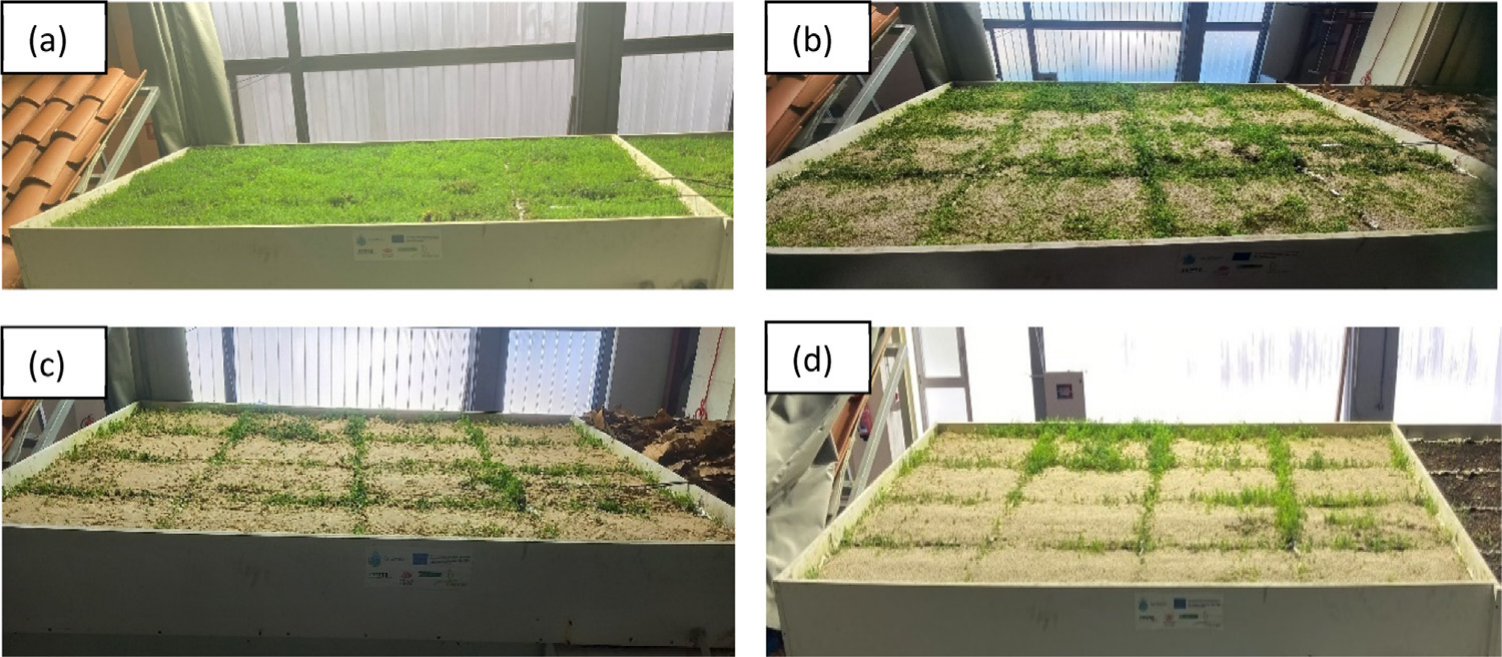
Sand accumulation scenario in Box 1. Total sand amount [kg.m^−2^]: (a) 2; (b) 6; (c) 14; (d) 18.

The sand accumulation scenario represented incremental BGI surface/substrate clogging. Sand with a median particle size distribution (d_50_) of 0.3 mm (range: 0.063 - 2 mm) was used to conduct the sand accumulation tests. Different amounts of sand (2, 4, 8, 4 kg.m^−2^) were added one test after another on Box 1 such that the cumulative sums for the sequential tests were 2, 6, 14, and 18 kg.m^−2^, respectively (Fig. 7). To ensure uniform distribution, the sand was spread using a mesh during each addition.

Once a specific amount of “dry” sand was added, the rainfall event (16.66 mm.h^−1^ for 30 min) was supplied, and the hydrological response was recorded. Then, more sand was added for another iteration after a delay of at least 24 hours to allow the soil and sand to dry. For some weights, the “dry” sand tests were followed up with a “wet” one in which another test was run after approximately four hours from the first test (by which time the underdrain flow had already ceased) without adding any more sand.

#### 4.3.3 Leaf litter accumulation

The leaf litter accumulation scenario depicted incremental BGI surface sealing. The leaves for the leaf litter experiment were collected at the premises of UDC and comprised broad-type leaves (*Platanus x hispanica*). The dimensions (length and width) of thirty randomly selected leaves were 10.8 ± 2.1 cm and 12.2 ± 2.6 cm, respectively. Dry leaves had a moisture content of 13-20% by weight; for wet leaves, it varied between 50 and 53%.

**Fig. 8:**
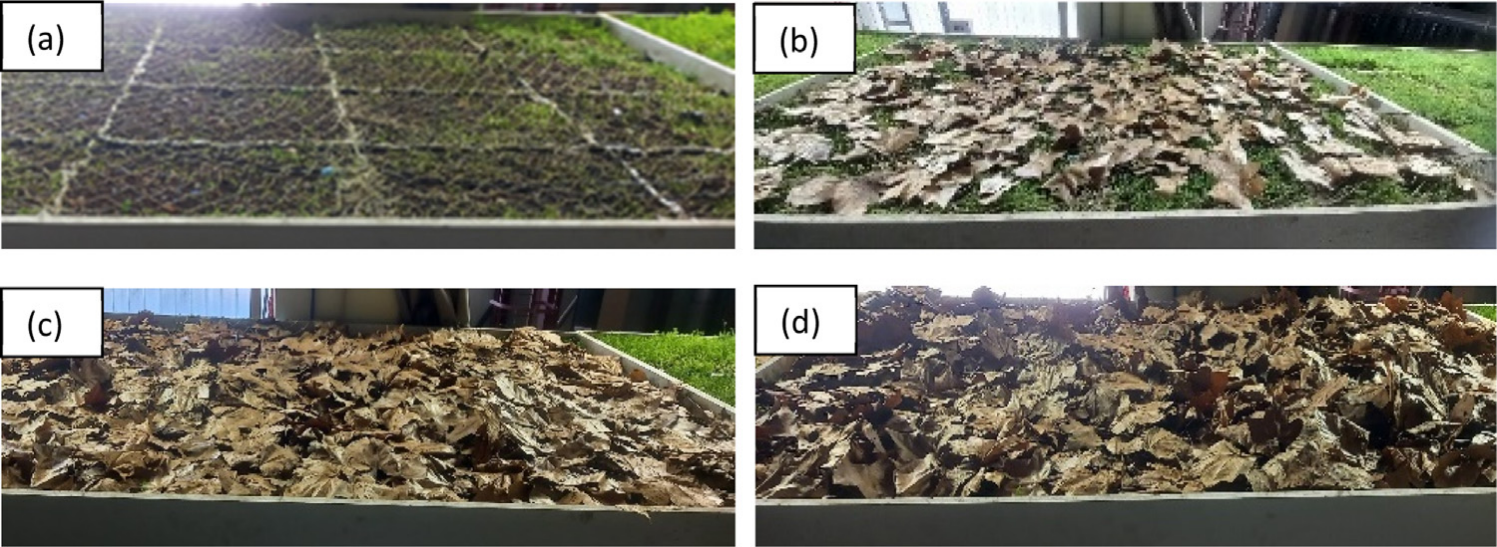
Incremental leaf litter in Box 2. Total leaves amount [kg]: (a) No litter (reference condition); (b) 0.3; (c) 1.2; (d) 1.725.

Leaves were added as 0.3, 1.2, and 1.725 kg (total), or ∼0.08 - 0.45 kg.m^−2^, and distributed randomly –albeit uniformly– across the BGI surface of Box 2 (18.66 mm.h^−1^ for 30 min) (Fig. 8). Once a specific amount of “dry” leaves was added, the rainfall event was supplied and the hydrologic response recorded. Following the "dry" test, another test was subsequently performed (after four hours) with the same amount of leaves, such that the performance for the "wet" leaves could also be recorded.

## Limitations

Despite the considerable insights provided by the data, several challenges were encountered during their procurement and processing. The experimental design inherently restricted the scope of the data to shock events, making it unsuitable for analysing the gradual, long-term deterioration of BGI. Furthermore, the quantities of sand and leaves introduced to simulate poor maintenance conditions were selected arbitrarily, ranging from mild to extreme levels, which may not fully represent real-world scenarios. Another limitation lay in testing a single type of rainfall event, which precluded the applicability of the findings to precipitation events with varying durations, intensities, and volumes. In addition, the nature of the experimental setup restricted the possibility of conducting redundant replicates, thereby limiting the number of available data.

## Ethics Statement

The authors have read and followed the ethical requirements for publication in Data in Brief and confirm that the current work does not involve human subjects, animal experiments, or any data collected from social media platforms.

## CRediT authorship contribution statement

**Prabhat Joshi:** Conceptualization, Data curation, Formal analysis, Investigation, Methodology, Project administration, Software, Validation, Visualization, Writing – original draft. **Juan Naves:** Investigation, Methodology, Supervision, Validation, Writing – review & editing. **Jose Anta:** Funding acquisition, Investigation, Project administration, Resources, Supervision, Validation, Writing – review & editing. **Max Maurer:** Conceptualization, Methodology, Resources, Supervision, Validation, Writing – review & editing. **João P. Leitão:** Conceptualization, Funding acquisition, Methodology, Project administration, Resources, Supervision, Validation, Writing – review & editing.

## Data Availability

ERIC/OpenDataset on the impacts of sand and leaf litter on the hydrological performance of green roofs as surrogate for infiltration-based blue-green infrastructure (BGI) (Original data).

## Footnotes

i CITEEC: https://www.udc.es/gl/citeec/hidraulica-en.html.
